# Supplementary material for: Optimising broadband pulses for DEER depends on concentration and distance range of interest
Source: Magn Reson (Gott). 2020 May 12;1(1):59–74. doi: 10.5194/mr-1-59-2020 (PMC10500711; doi:10.5194/mr-1-59-2020)
Supplement: The supplement related to this article is available online at: https://doi.org/10.5194/mr-1-59-2020-supplement. [file mr-1-59-supplement.pdf]

Supplement of Magn. Reson., 1, 59–74, 2020
https://doi.org/10.5194/mr-1-59-2020-supplement
© Author(s) 2020. This work is distributed under
the Creative Commons Attribution 4.0 License.

Open Access
MAGNETIC RESONANCE

Supplement of

Optimising broadband pulses for DEER depends on concentration and distance range of interest

Andreas Scherer et al.

Correspondence to: Malte Drescher (malte.drescher@uni-konstanz.de)

The copyright of individual parts of the supplement might differ from the CC BY 4.0 License.

Contents

S1 Additional materials and methods	3
S2 The MNR as the function of merit	5
S3 Determination of the integration window	7
S4 Parameters for the observer pulse	9
S5 The MNR for rectangular and Gaussian pump pulses evaluated up to 7 µs	10
S6 Inversion profiles for rectangular and Gaussian pulses	11
S7 Simulations of spin inversion trajectories	12
S8 The MNR for broadband pump pulses evaluated up to 7 µs	13
S9 Inversion profiles for the broadband shaped pulses	15
S10 Pulse shapes of the broadband shaped pulses	16
S11 Comparison of simulated and experimental inversion profile	17
S12 Full DEER traces	18
S13 The influence of the length of broadband shaped pump pulses	19
S14 The influence of the B_1 field strength on chirp pulses	21
S15 Comparison of bandwidth compensated and non-bandwidth compensated pulses	21
S16 The MNR for rectangular and Gaussian pump pulses evaluated up to 2 µs	22
S17 The MNR for broadband pump pulses evaluated up to 2 µs	23
S18 The MNR of the diluted sample evaluated up to 7 µs	25
S19 Correlation between the background density and the modulation depth	25
S20 Background of the DEER traces	26
S21 Calculation of the background-dependent performance of broadband shaped pulses	27
S22 Comparison of the resonator profiles	29
S23 Supporting Information References	29

S1 Additional materials and methods

All experiments have been performed on a Bruker Elexsys E580 spectrometer at Q-band (34 GHz). The spectrometer is equipped with a SpinJet-AWG unit (Bruker) and a 150 W pulsed travelling-wave tube (TWT) amplifier (Applied Systems Engineering, Fort Worth, USA). All samples were measured in 3 mm outer diameter sample tubes in an overcoupled ER5106QT-2 resonator (Bruker). The quality factor Q of the overcoupled resonator is approximately 200.

The samples were cooled to 50 K with a Flexline helium recirculation system (CE-FLEX-4K-0110, Bruker Biospin, ColdEdge Technologies) comprising a cold head (expander, SRDK-408D2) and a F-70H compressor (both SHI cryogenics, Tokyo, Japan), controlled by an Oxford Instruments Mercury ITC.

S1.1 EDFS

The echo-detected-field sweep spectra were recorded with a Hahn echo sequence ($\frac{\pi}{2}$ - τ - π - $\tau -$ echo) pulse-sequence with $\tau = 1.5$ µs, a sweep width of 200 G and 10 shots per point, 3 scans and rectangular pulses. The length of the π-pulse was 16 ns at a frequency of 34 GHz.

S1.2 Nutation experiments

Pulse lengths of rectangular and Gaussian pulses were determined with nutation experiments with the pulse sequence (inversion pulse $- \tau_1 - \frac{\pi}{2} - \tau_2 - \pi - \tau_2 -$ echo). τ_1 was set to 1 µs.

S1.3 Resonator profile

The resonator profile was measured by a series of nutation experiments at different frequencies as described in the literature (Doll and Jeschke, 2014). It was measured over 300 MHz with a step size of 10 MHz. The magnetic field was co-stepped. The nutation frequencies were calculated by a Fourier transformation of the nutation traces.

S1.4 DEER

All DEER experiments were measured with the standard four pulse DEER sequence:

$$\frac{\pi}{2_{obs}} - \tau_1 - \pi_{\mathrm{obs}} - t - \pi_{\mathrm{pump}} - \tau_1 + \tau_2 - t - \pi_{\mathrm{obs}} - \tau_2 - \mathrm{echo}$$

The delay between the $\pi/2$ and the π pulse in the observer channel τ_1 was 400 ns. The dipolar evolution time τ_2 was 8 µs. For all DEER experiments with rectangular and Gaussian pump pulses the pump frequency was set to 34.00 GHz. The magnetic field was chosen such that the pump lies on the maximum of the nitroxide spectrum. We used the phase cycling ((x) [x] x_p x) as suggested by (Tait and Stoll, 2016) and nuclear modulation averaging as suggested by (Jeschke, 2012).

S1.5 DEER optimisation

For the optimisation measurements we used a python script that can automatically perform several DEER experiments after another. We shifted the magnetic field from 1.2090 T to 1.2113 T for an observer pulse of 33.91 GHz and from 1.2097 T to 1.2119 T for an observer position of 33.93 GHz to ensure that the pump pulse is on the maximum of the nitroxide spectrum. Figure S1 illustrates the idea with a fixed observer frequency of 33.93 GHz.

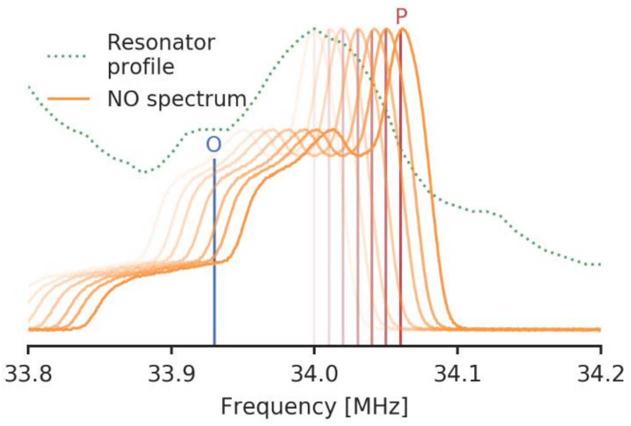

Figure S1: The resonator profile (green dots) with the different offsets during an optimisation measurement. The nitroxide spectrum (orange) is shifted with the offset. The observer frequency stays fixed at 33.93 GHz and is indicated by a blue line. The shift of the pump spin is indicated by the red line.

S1.6 Pulse calculations

For rectangular and Gaussian pulses, we used the pulses that are generated by Bruker Xepr software. For Gaussian pulses the FWHM is defined by FWHM $= \frac{t_{\mathrm{p}}}{2\sqrt{2\ln(2)}}$. All other pulses were calculated with the *pulse* function from the *easyspin* (Version 5.2.21) package for MATLAB R2018b (Stoll and Schweiger, 2006). The resulting pulse shapes were normalised to amplitude values between -1 and 1 and loaded into Xepr.

S1.7 Integration window

The integration window was determined by recording a series of 300 Hahn echoes in transient mode. We evaluated the signal-to-noise (SNR) with different integration windows and determined the integration window with the maximum SNR.

S1.8 Inversion profiles

Inversion profiles for broadband shaped pulses were measured with the pulse sequence.

$$\text{broadband shaped pulse} - \tau_1 - \frac{\pi}{2}_{obs} - \tau_2 - \pi_{\text{obs}} - \tau_2 - \text{echo}$$

The inversion profiles were measured as the echo intensity as function of the frequency offset of the initial broadband shaped pulses. The $\pi/2$ and π pulses were rectangular pulses with a fixed frequency of 34 GHz.

S2 The MNR as the function of merit

Here, we want to discuss whether the MNR is a suitable function of merit for the determination of distance distributions and up to which time point in the DEER trace, the MNR needs to be evaluated to serve this purpose. Therefore, we performed simulations with a model distance distribution p_0 that is based on the narrow distance distribution of the model system used in this study. We approximated the experimentally obtained distance distribution with a Gaussian with a mean at 5.08 nm and a standard deviation of 0.08 nm. We varied the background density in ten steps from k = 0.01 1/μs to k = 0.3 1/μs in combination with a low, medium and high noise level (noise σ_0 = 0.02, 0.05 and 0.1) that was added to the DEER trace. The background dimension was set to d = 3 and a modulation depth of 0.5 was used. The DEER traces were simulated in the time domain up to 8 μs. For each parameter set we generated ten different traces. To compare the background correction by division (Jeschke et al., 2006) with the kernel inclusion approach as described in (Fábregas Ibáñez and Jeschke, 2020) we analysed all simulated DEER traces with both methods. We did not fit the background but used the true background function. The regularisation parameter was chosen according to the generalised cross-validation method. The quality of the resulting distance distributions p was estimated by the Euclidian distance D from the true distance p_0:

$$D(p, p_0) = \|p - p_0\|_2 \quad (1)$$

The MNR of the form factor F was calculated as described in the main text up to a limit of 7 μs according to equation (13) of the main text.

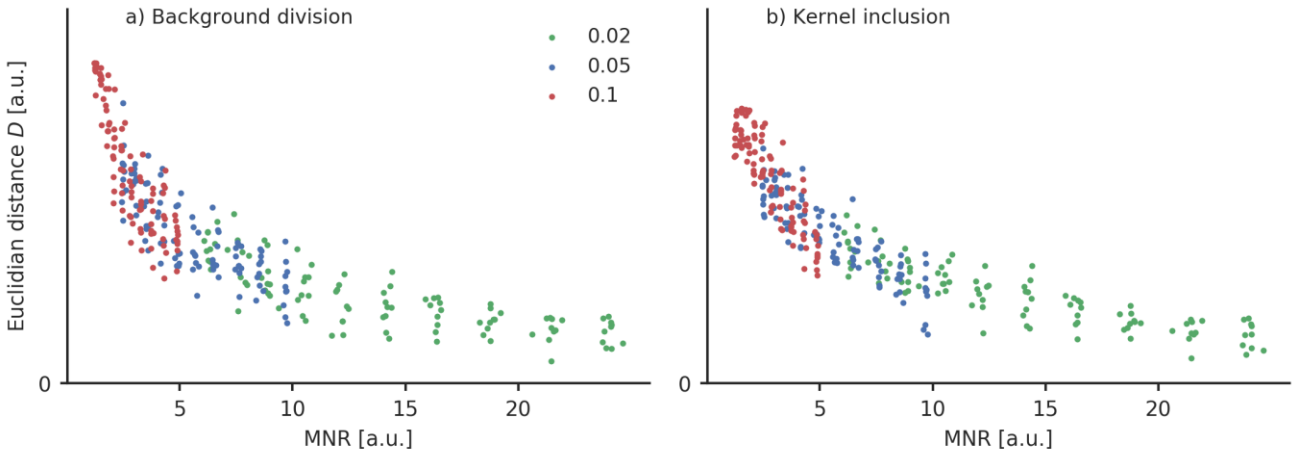

Figure S2: The Euclidian distance D of the real and calculated distance distribution as defined in equation (1) is plotted as a function of the MNR. Each dot represents a simulated DEER trace with either low (σ_0 = 0.02, green), medium (σ_0 = 0.05, blue) and high (σ_0 = 0.1, red) noise. The background correction was performed by (a) dividing the DEER trace by the background and (b) including the background in the kernel.

In Fig. S2, the quality of the determined distance distribution was plotted as a function of the determined MNR for both a background correction by division (Fig. S2a) and a kernel inclusion approach (Fig. S2b). For each noise level the MNR only depends on the density of the background as all other parameters are kept constant and only the background density is varied. So a lower MNR corresponds to a higher background density rate and vice versa. For the low noise level (σ_0 = 0.02), the quality of the determined distance distributions only varies a little for different background density rates. For medium (σ_0 = 0.05) and high (σ_0 = 0.1) noise levels, however, the dependency of the quality of the determined distance distribution decreases significantly with a decreasing MNR. If the MNR is only evaluated up to an early point of the form factor, the information of the background decay rate is lost in this case and is not properly included in the MNR as the MNR would then depend nearly exclusively on the given noise level.

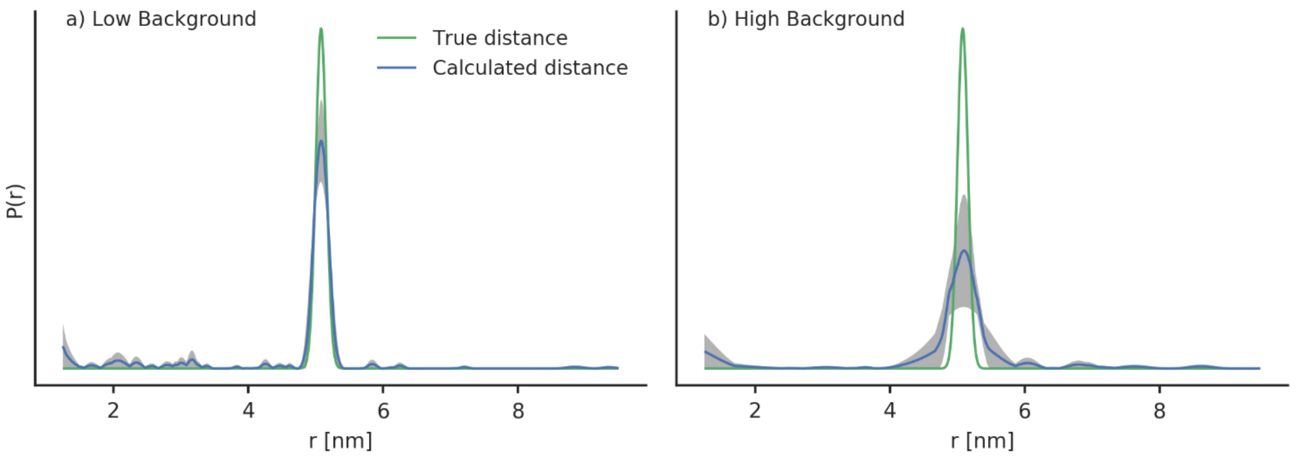

Figure S3: An exemplary distance distribution obtained for a medium noise level (σ_0 = 0.02) with (a) a low background density (k = 0.01 MHz) and (b) a high background density (k = 0.3 MHz). The grey area shows the area that is covered by the calculated distance distribution for ten exemplary DEER traces. The mean of the shaded area is drawn in blue and the true distance is drawn in green.

A closer inspection reveals that whereas the obtained distance distributions for high background densities reproduce the mean of the distance distribution correctly, they overestimate the width of the distribution and the distance appears to be broader as it is (see Fig. S3 for an exemplary data set). Depending on the information that shall be obtained by the DEER measurements, the mean of the distance distribution might suffice. However, if high resolution distance distributions shall be obtained, it seems to be important to optimise the MNR up to the limit which is given by equation (13) of the main text. The comparison of both background correction methods shows that the kernel inclusion gives better results particularly for a high noise and a high background decay. It should therefore be considered as the superior method. However, the correlation between the quality of the determined distance distribution and the MNR is still valid. This is why, we consider the MNR as a proper function of merit, even if the kernel inclusion approach is used.

For a more comprehensive study, the effect of the MNR on the quality of the obtained distance distribution could also be tested for distance distributions with different distance ranges and widths. Such a detailed study was, however, beyond the scope of the this manuscript.

S3 Determination of the integration window

To determine the ideal integration window we recorded a series of Hahn echoes and calculated the SNR ratio for different integration window lengths. The results show that for rectangular pulses the ideal integration window is typically longer than the π-pulse length (Fig. S4). An improvement of up to 14 % for a π-pulse length of 28 ns and an ideal integration window of 44 ns was achieved.

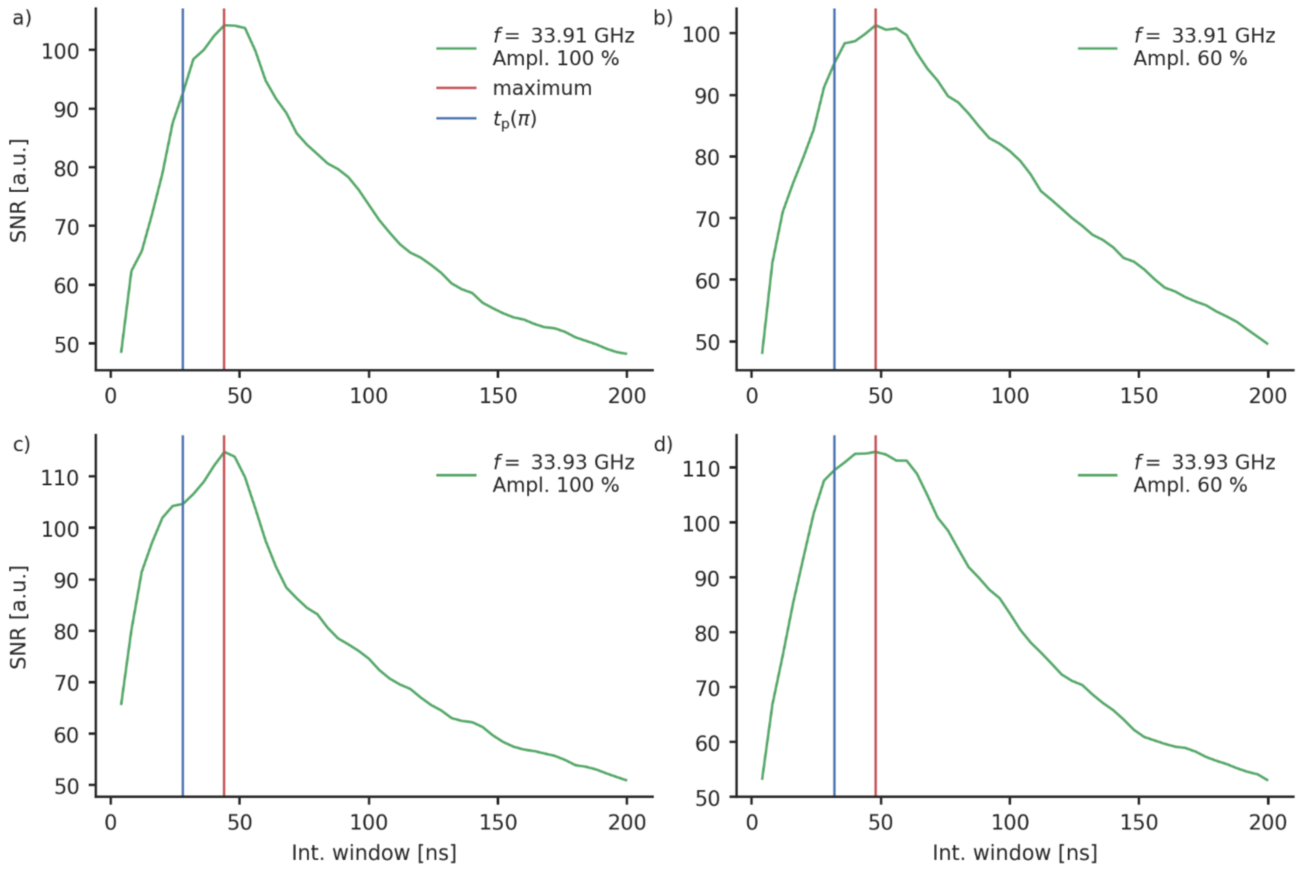

Figure S4: The SNR for rectangular pulses of a series of transient Hahn echoes is shown as a function of the integration window length. The red lines indicate the integration window with the maximum SNR, the blue lines indicate an integration window that has the length of the π-pulse. The pulses have the following settings: a) frequency: 33.91 GHz, amplitude: 100 %. b) frequency: 33.91 GHz, amplitude: 60 %. c) frequency: 33.93 GHz, amplitude: 100 %. d) frequency: 33.93 GHz, amplitude: 60 %.

For Gaussian pulses the ideal integration window is typically smaller than the π-pulse length (Fig. S5).

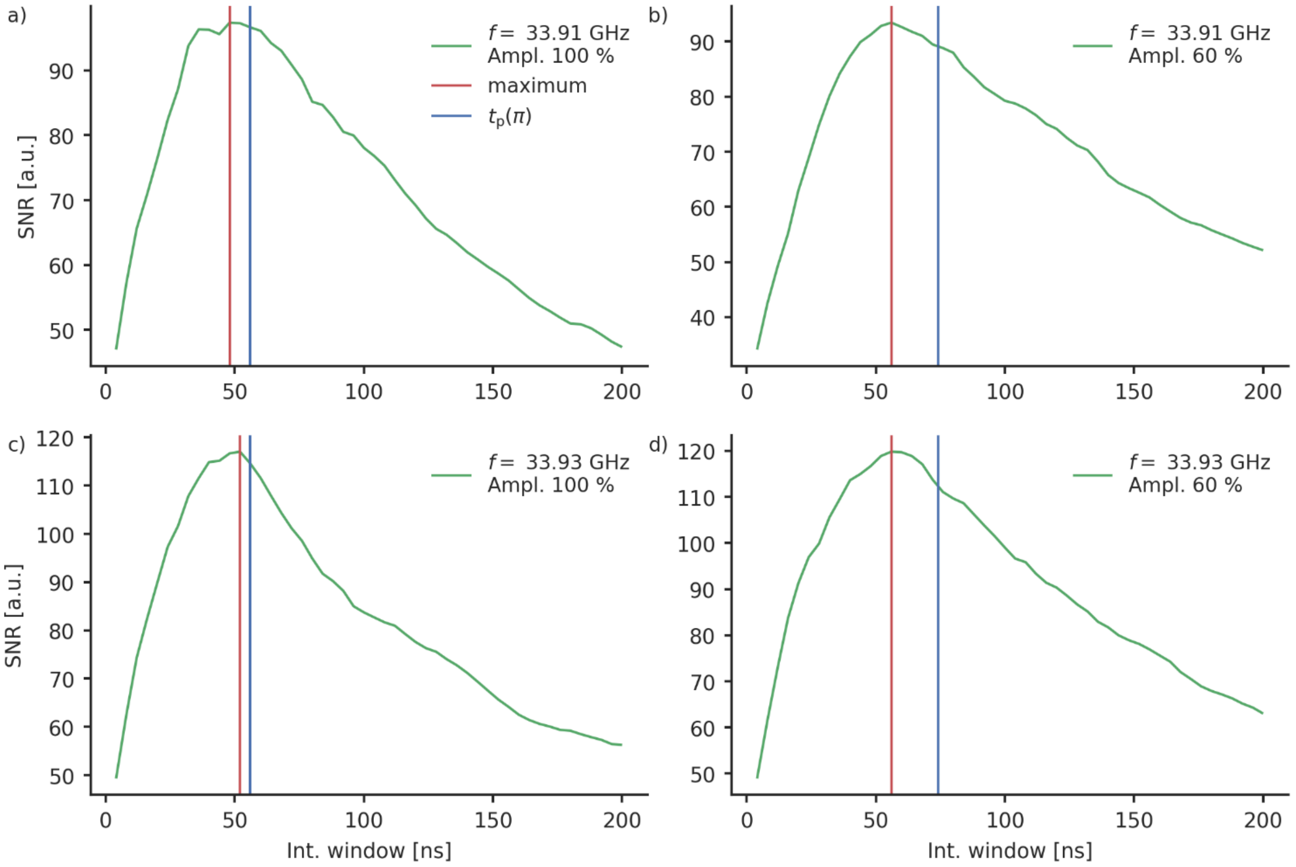

Figure S5: The SNR for Gaussian pulses of a series of transient Hahn echoes is shown as a function of the integration window length. The red lines indicate the integration window with the maximum SNR, the blue lines indicate an integration window that has the length of the π-pulse. The pulses have the following settings: a) frequency: 33.91 GHz, amplitude: 100 %. b) frequency: 33.91 GHz, amplitude: 60 %. c) frequency: 33.93 GHz, amplitude: 100 %. d) frequency: 33.93 GHz, amplitude: 60 %.

S4 Parameters for the observer pulse

Table S1: Parameters for the rectangular observer pulses. The pulse length is referring to the π-pulse.

f_{obs} [GHz]	Obs. Amp. [%]	t_π [ns]	Length of integration window [ns]
**33.91**	100	28	44
	60	32	48
**33.93**	100	28	44
	60	32	48

Table S2: Parameters for the Gaussian observer pulses. The pulse length is referring to the π-pulse.

f_{obs} [GHz]	Obs. Amp. [%]	t_π [ns]	Length of integration window [ns]
**33.91**	100	56	48
	60	74	56
**33.93**	100	56	52
	60	74	56

S5 The MNR for rectangular and Gaussian pump pulses evaluated up to 7 µs

Table S3: MNR for a rectangular pump pulse and different rectangular observer pulses. The pump pulses had a length of 16 ns. The MNR has been evaluated up to 7 µs.

f_{obs} [GHz]	Obs. Amp. [%]	t_π [ns]	MNR	Mod depth λ
33.91	100	28	30	0.32
	60	32	32	0.32
33.93	100	28	32	0.31
	60	32	35	0.31

Table S4: MNR for a Gaussian pump pulse and different Gaussian observer pulses. The pump pulses had a length of 34 ns. The MNR has been evaluated up to 7 µs.

f_{obs} [GHz]	Obs. Amp. [%]	t_π [ns]	MNR	Mod depth λ
33.91	100	56	36	0.31
	60	74	32	0.29
33.93	100	56	41	0.31
	60	74	38	0.31

S6 Inversion profiles for rectangular and Gaussian pulses

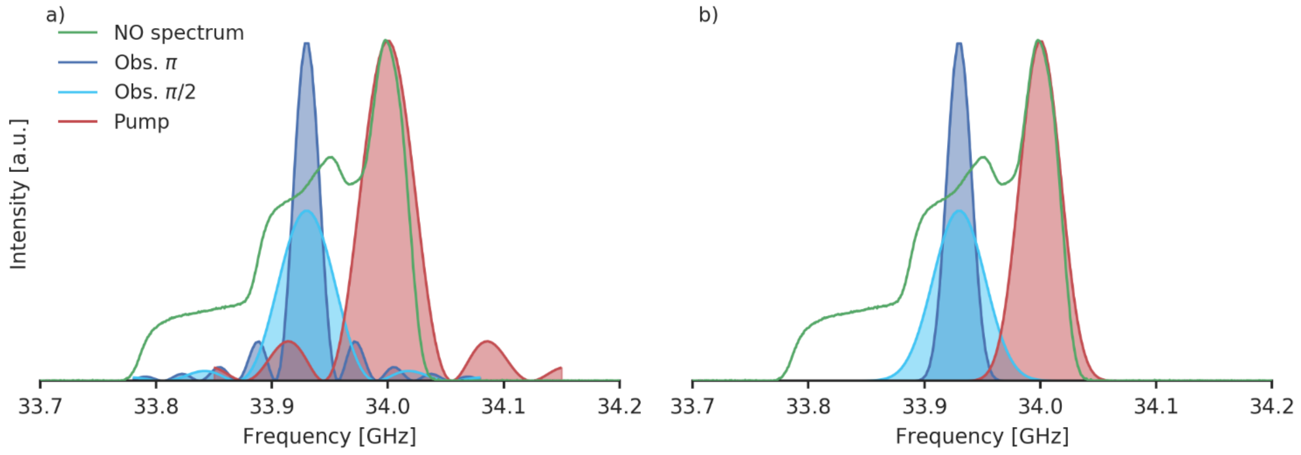

Figure S6: The excitation profiles of the observer (blue) and pump pulses (red) of (a) rectangular and (b) Gaussian pulses. The light blue profiles are for the $\pi/2$ observer pulses and the dark blue profiles for the π observer pulse. The rectangular observer pulses have an amplitude of 60 %, a pulse length of 32 ns (π on observer) and 16 ns (pump), and the Gaussian have an amplitude of 100 %, a length of 56 ns (π on observer) and 34 ns (pump). It can be seen that the spectral overlap can be reduced with Gaussian pulses. The pulse amplitudes of the pump pulse are always 100 %.

S7 Simulations of spin inversion trajectories

We simulated the effect of an HS{1,1} pulse with a pulse length of 100 ns, a truncation parameter of $\beta = 8/t_p$, a frequency sweep range from -55 MHz and 55 MHz. We performed the numerical simulation in the density operator framework with MATLAB R2018b. The maximum of the B_1-field was set to 30 MHz, which corresponds to the maximum of the resonator profile. This pulse shows a good inversion between a frequency range of approximately -40 MHz and 40 MHz (Fig. S7a). In Fig. S7b, the inversion of a spin packet with an offset of -40 MHz and 40 MHz are shown. It can be seen that the spins are inverted in a time window between roughly 20 ns and 80 ns, making an effective pulse length of 60 ns. This would correspond to a minimum distance of 2.32 nm. Note that these numbers were only obtained by visual inspection, so this should only be considered as a qualitative discussion. The spin flip behaviours is also different for different pulses.

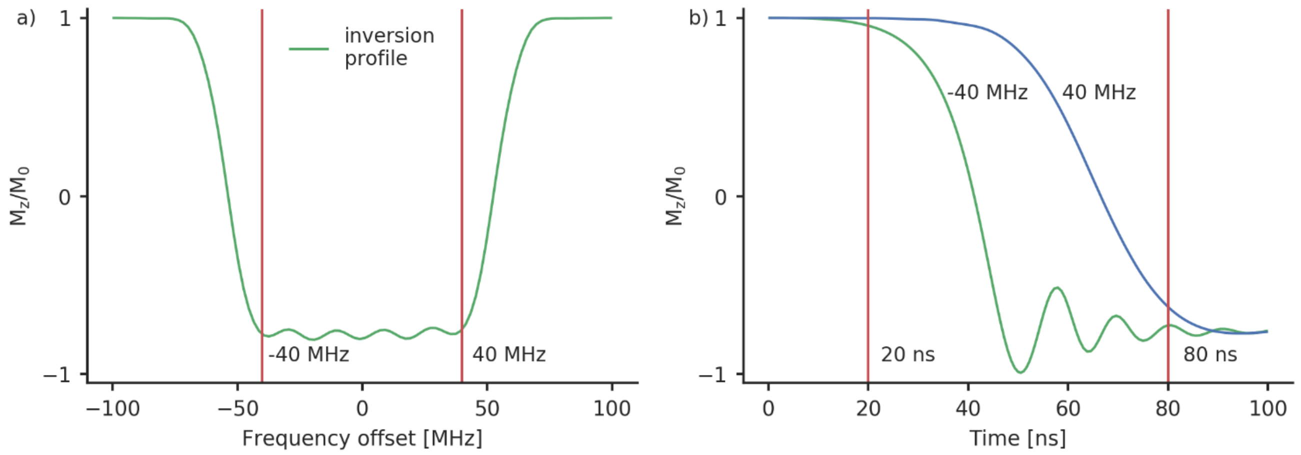

Figure S7: a) The excitation profile of HS{1,1} with a pulse length of 100 ns, a truncation of $\beta = 8/t_p$ and a frequency range from -55 MHz and +55 MHz. b) The inversion of a spin packet with an offset of -40 MHz (blue) and +40 MHz (green).

S8 The MNR for broadband pump pulses evaluated up to 7 μs

Table S5: MNR for the different broadband shaped pulses with a rectangular observer pulse. The MNR has been evaluated up to 7 μs.

f_{obs} [GHz]	Obs. Amp. [%]	Pump pulse	t_π [ns]	Δf [MHz]	Offset [MHz]	MNR	Mod. depth λ
**33.91**	100	HS{1,6} ($\beta = 8/t_p$)	100	110	90	32	0.63
		WURST (n=6)	100	160	90	37	0.64
		Chirp ($t_r = 30$ ns)	36	120	90	33	0.50
		HS{1,1} ($\beta = 6/t_p$)	100	110	90	41	0.57
	60	HS{1,6} ($\beta = 10/t_p$)	100	110	90	31	0.62
		WURST (n=6)	100	160	90	30	0.64
		Chirp ($t_r = 30$ ns)	100	160	90	32	0.67
		HS{1,1} ($\beta = 6/t_p$)	100	110	90	34	0.57
**33.93**	100	HS{1,6} ($\beta = 10/t_p$)	100	110	100	39	0.57
		WURST (n=6)	100	160	100	39	0.62
		Chirp ($t_r = 10$ ns)	36	120	90	38	0.47
		HS{1,1} ($\beta = 6/t_p$)	100	110	90	43	0.53
	60	HS{1,6} ($\beta = 10/t_p$)	100	110	90	40	0.60
		WURST (n=6)	100	120	100	36	0.57
		Chirp ($t_r = 10$ ns)	36	120	90	38	0.47
		HS{1,1} ($\beta = 8/t_p$)	100	90	80	43	0.48

Table S6: MNR for the different broadband shaped pulses with a Gaussian observer pulse. The MNR has been evaluated up to 7 µs.

f_{obs} [GHz]	Obs. Amp. [%]	Pump pulse	t_π [ns]	Δf [MHz]	Offset [MHz]	MNR	Mod. depth λ
**33.91**	100	HS{1,6} ($\beta = 10/t_p$)	100	90	90	36	0.60
		WURST (n=6)	100	160	90	30	0.64
		Chirp ($t_r = 10$ ns)	36	120	80	37	0.50
		HS{1,1} ($\beta = 6/t_p$)	100	110	90	40	0.58
	60	HS{1,6} ($\beta = 8/t_p$)	100	110	90	38	0.63
		WURST (n=6)	100	160	100	34	0.48
		Chirp ($t_r = 10$ ns)	100	120	90	38	0.48
		HS{1,1} ($\beta = 6/t_p$)	100	110	90	37	0.58
**33.93**	100	HS{1,6} ($\beta = 10/t_p$)	100	90	90	39	0.57
		WURST (n=6)	100	160	100	38	0.62
		Chirp (no smoothing)	36	120	80	45	0.49
		HS{1,1} ($\beta = 8/t_p$)	100	110	90	50	0.52
	60	HS{1,6} ($\beta = 10/t_p$)	100	110	90	45	0.61
		WURST (n=6)	100	160	90	40	0.63
		Chirp ($t_r = 9$ ns)	36	120	80	45	0.47
		HS{1,6} ($\beta = 8/t_p$)	100	110	80	47	0.52

S9 Inversion profiles for the broadband shaped pulses

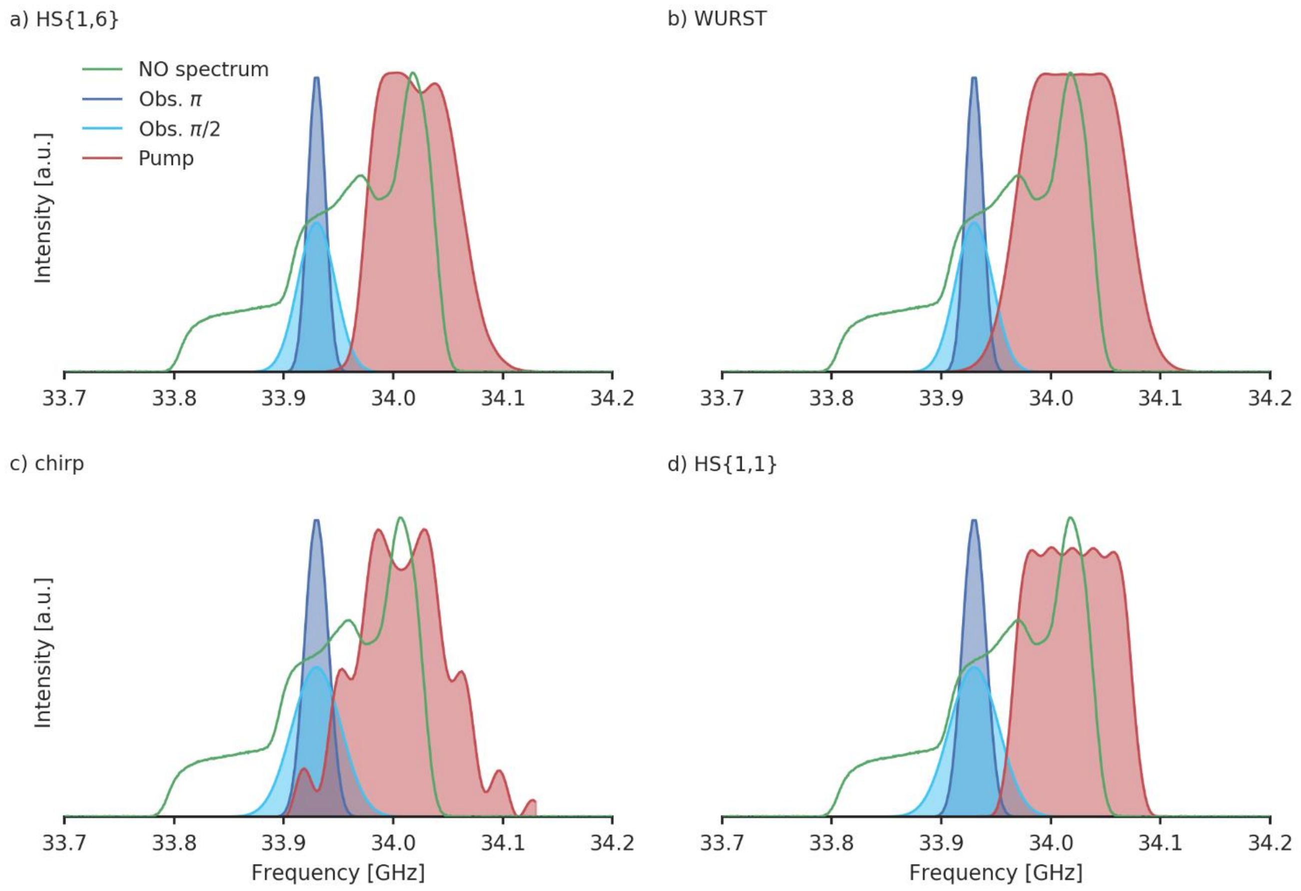

Figure S8: The excitation profiles of the best performing (a) HS{1,6}, (b) WURST, (c) chirp and (d) HS{1,1} pulse. The parameters of the pump and observer pulses can be found in table 3 of the main text.

S10 Pulse shapes of the broadband shaped pulses

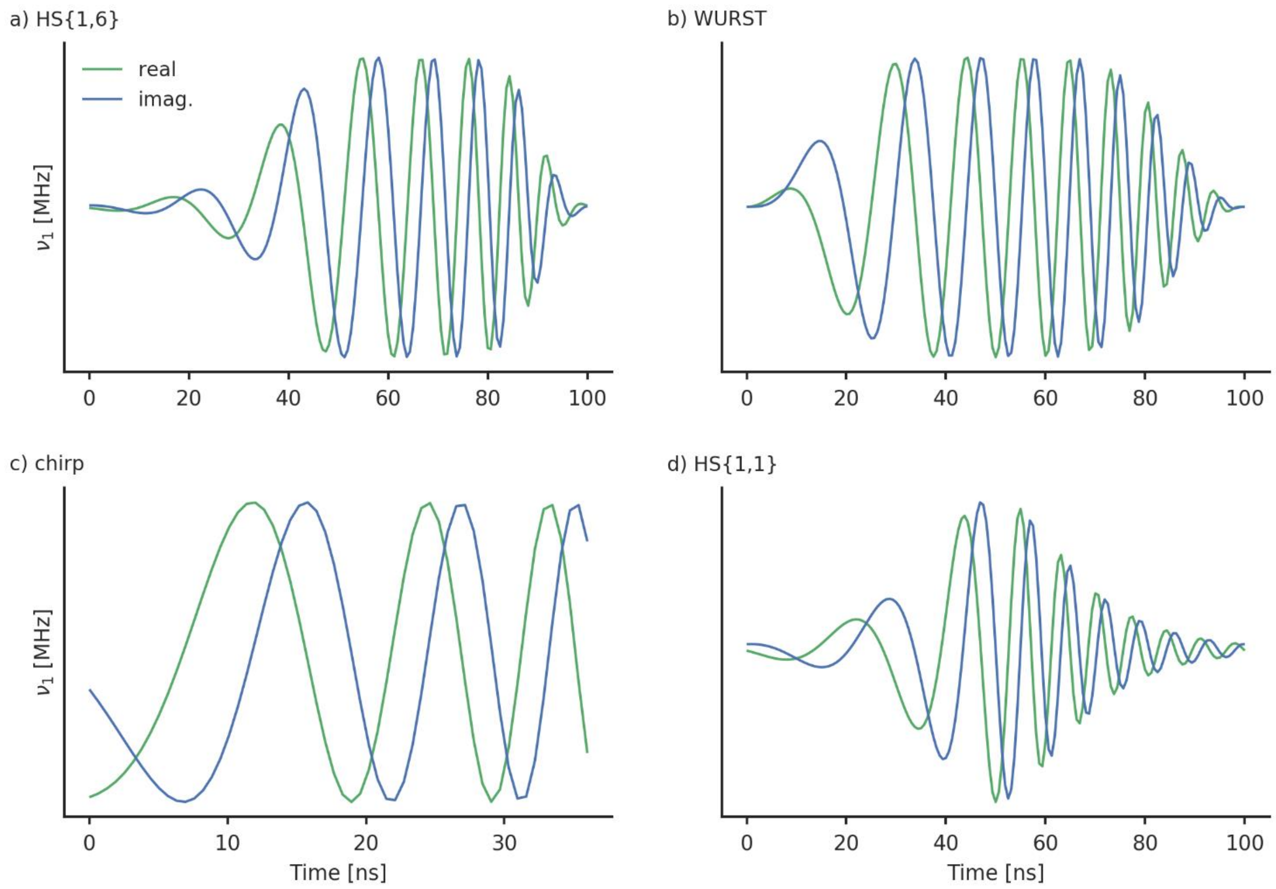

Figure S9: The pulse shapes of the best performing (a) HS{1,6} , (b) WURST , (c) chirp and (d) HS{1,1} pulse with the real part (green) and imaginary part (blue). The parameters broadband shaped pulses can be found in table 3 of the main text.

S11 Comparison of simulated and experimental inversion profile

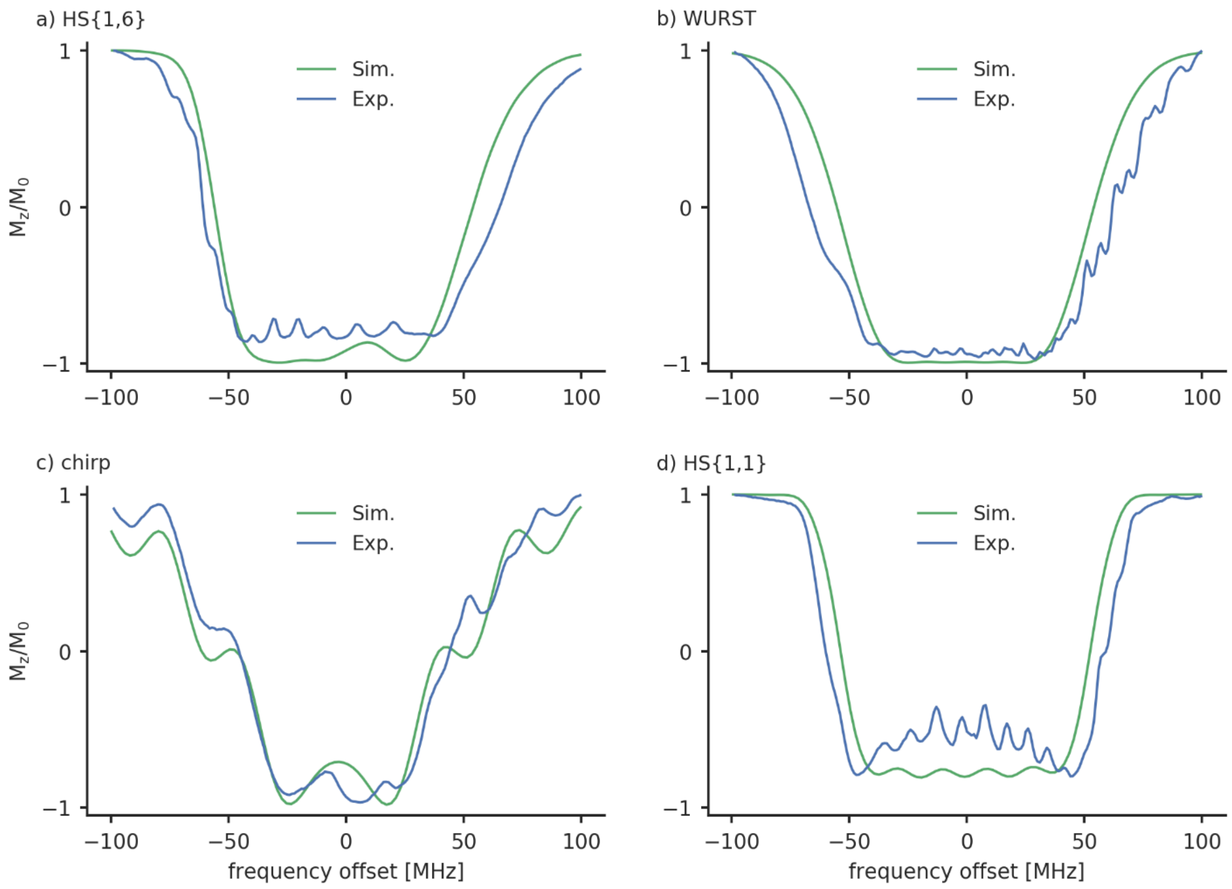

Figure S10: The simulated (green) and experimentally recorded (blue) inversion profiles of the best performing (a) HS{1,6}, (b) WURST, (c) chirp and (d) HS{1,1} pulse. The parameters of the pump and observer pulses can be found in table 3 of the main text.

We recorded the inversion profiles of the best performing pulses and compared them with the simulations in order to detect potential deviations. The results in Fit. S10 shows that the experimentally recorded inversion profiles reproduce the general trends of the simulations. Nonetheless, there are some deviations that are probably caused by the spectromeer and that shall be discussed here.

It can be noticed that for HS{1,6} and HS{1,1} pulses the measured inversion profiles do not reach the inversion profile of the simulation. For HS{1,6} the overall inversion efficiency is a bit lower than expected and for HS{1,1} pulses a bump in the centre of the frequency sweep was found. The simulations can, however, predict the fact that HS{1,6} have a higher inversion efficiency than HS{1,1} pulses. For WURST and chirp pulses the experimentally recorded inversion profiles reach the inversion efficiency of the simulation.

The experimental inversion profiles of HS{1,6}, HS{1,1} and chirp pulses have a larger inversion range than what is predicted by the simlations. This can increase the overlap with the observer pulse and therefore reduce the echo intensity. But as the inversion range is only a little bit larger, we consider this not to be particularly worrysome. For HS{1,6} and HS{1,1} pulses, the larger inversion range could compensate the reduced inversion efficiency

For the chirp pulses the frequency range as well as the inversion efficiency of the experimental and simulated inversion profiles agree. There are some minor deviations in the pattern of the oscillations that are present in the inversion profile, which we do not expect to have a large effect on the performance of the pulse.

S12 Full DEER traces

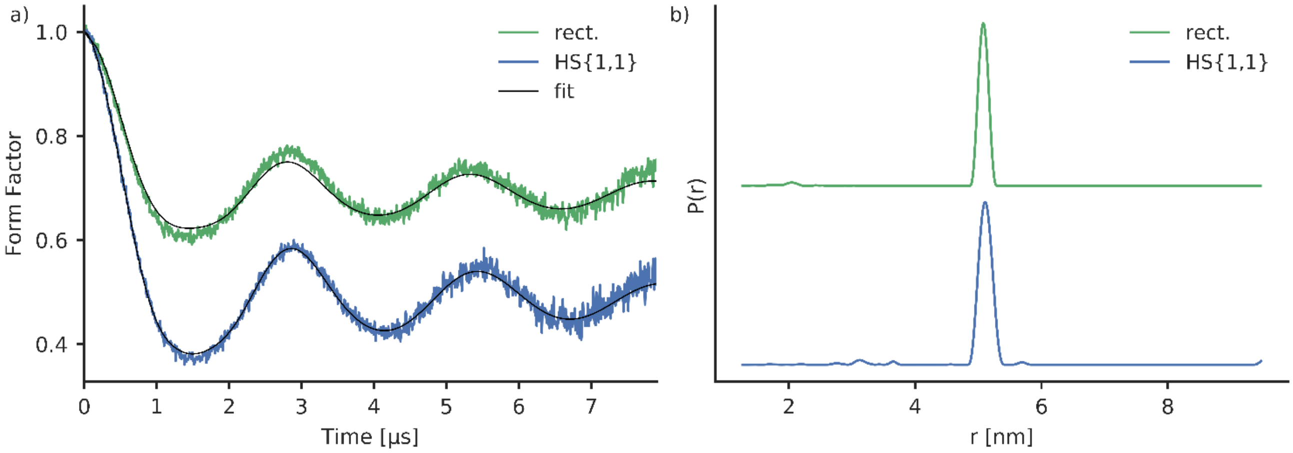

Figure S11: Comparison of the performance of DEER with rectangular pulses (green) and with Gaussian observer pulses and the HS{1,1} pulse from table 1 that yielded the best MNR (blue). The form factors are shown in (a) and the corresponding distance distributions in (b). One 10 minute scan was recorded for both experiments. The corresponding DEER traces are depicted in Fig. S17.

S13 The influence of the length of broadband shaped pump pulses

Tests with broadband shaped pump pulses with pulse lengths of 200 ns and 400 ns showed that they do not lead to an overall performance increase. This is shown here exemplary by comparing the performance of HS{1,1} pump pulses and Gaussian observer pulses (Fig. S12). There are indeed some pump pulses (for example a HS{1,1} pulse with $\beta = 10/t_p$ and $\Delta f = 110$ MHz) that show an improvement with a longer pulse length, however there is no overall gain by using a pump pulse length of 200 ns.

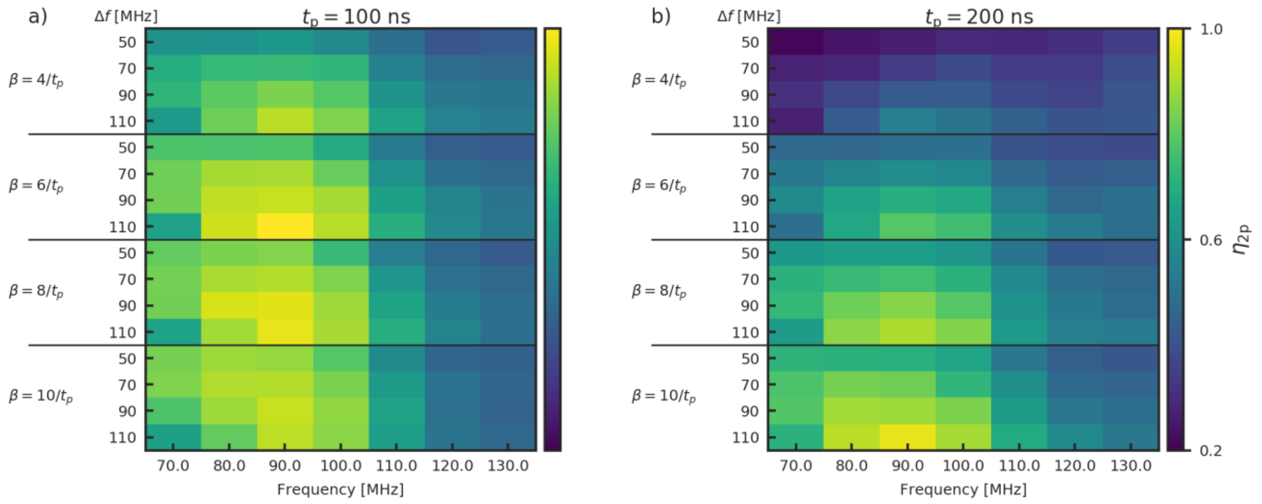

Figure S12: HS{1,1} pump pulses of (a) 100 ns and (b) 200 ns length. The observer pulses were Gaussian pulses with 100 % intensity at an observer position with a 90 MHz offset from the centre of the resonator profile and a pulse length of 56 ns for the π pulse. The colour bars are normalised to the same value so that both heat maps are comparable.

We noticed that a major problem with longer broadband shaped pump pulses is that the intensity of the echo can be reduced (Fig. S13a). For a pump pulse offset of 90 MHz, the echo intensity at the zero time of the DEER trace is reduced significantly when increasing the pump pulse lengths from 100 ns over 200 ns to 400 ns.

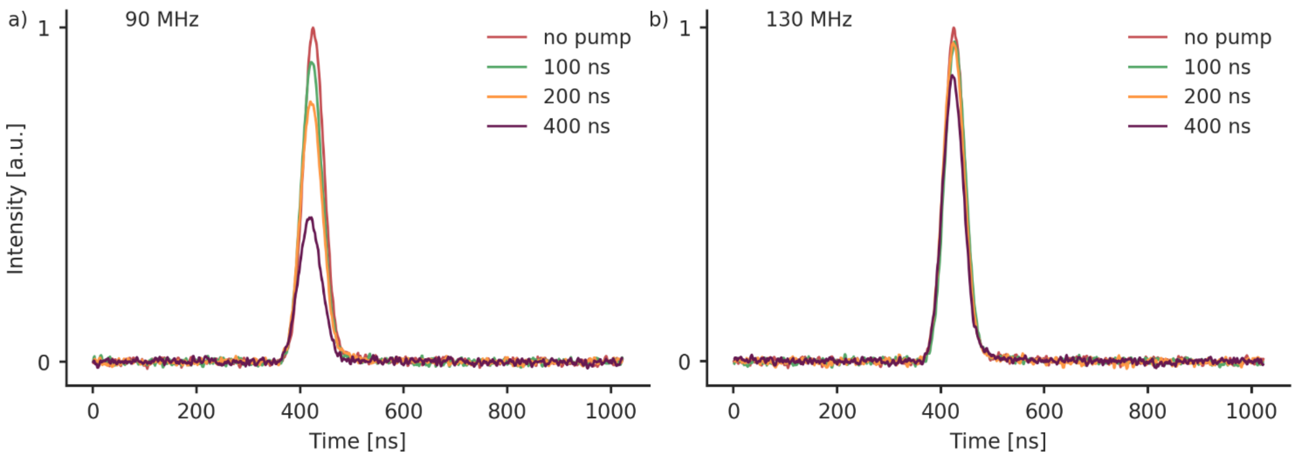

Figure S13: The echo at the zero time of the DEER trace. The observer pulses were Gaussian pulses with 100 % intensity at an observer position with a 70 MHz offset from the centre of the resonator profile and a pulse length of 56 ns for the π pulse. The pump pulses were HS{1,1} pulses with $\beta = 8/t_p$ and $\Delta f = 110$ MHz. The offset between the pulses is (a) 90 MHz and (b) 130 MHz.

A comparison of the calculated inversion profiles of the respective pulses (Fig. S14a-c) shows that, whereas the 100 ns pulse should lead to an incomplete inversion, a nearly complete inversion can be expected for the longer pulses. Furthermore, the longer pulses should have slightly steeper excitation flanks. Those trends can indeed be found for the measured inversion profiles. There are some deviations of the measured and calculated inversion profiles. The measured inversion profile of the 100 ns pulse shows an increased frequency width compared to the calculated profile. Furthermore, there is bump in the centre of the frequency sweep. The measured inversion profiles of the longer pulses show the expected steep frequency flanks that can also be seen in the simulation. The inversion profiles of the 200 ns and the 400 ns pulses show a small asymmetry around the centre of the frequency sweep. We assign these deviations to instrumental pulse distortions caused by the spectrometer.

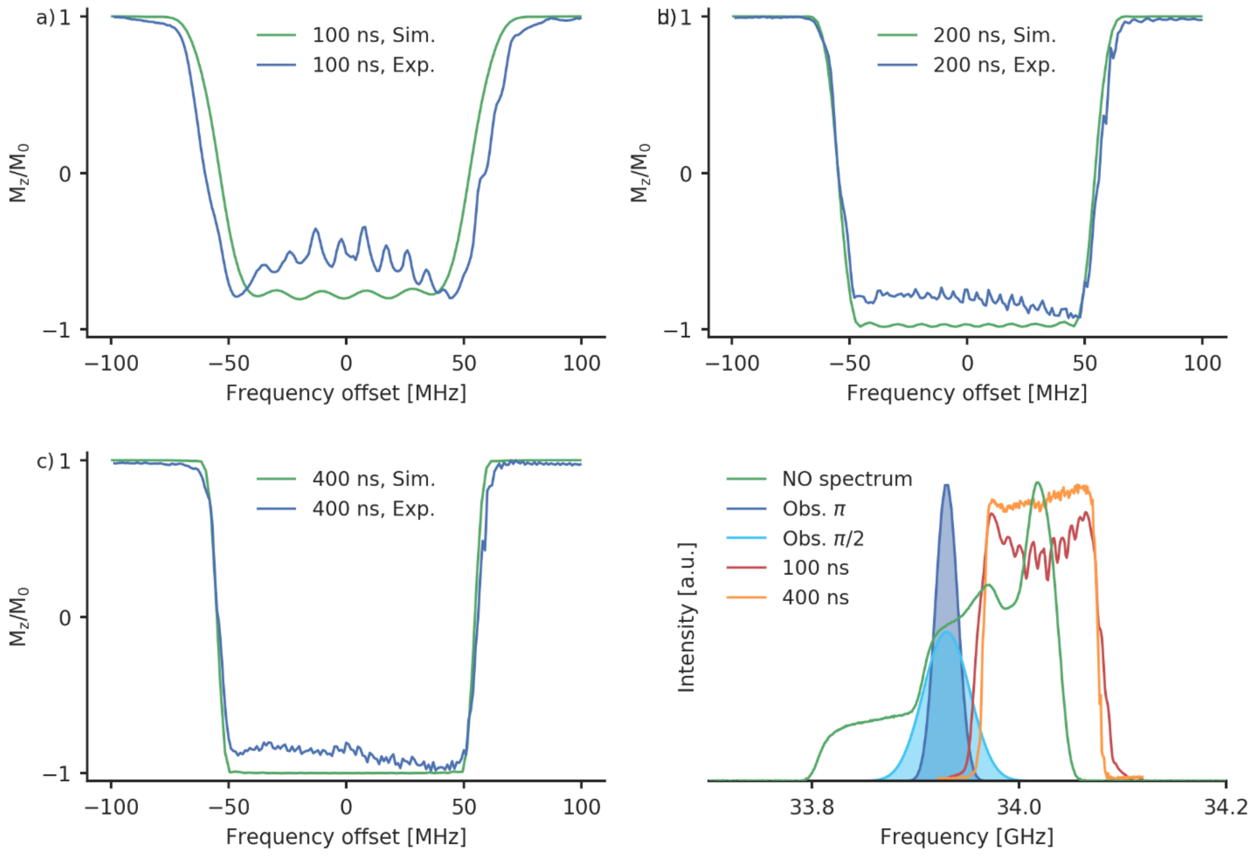

Figure S14: Calculated and measured inversion profiles of a HS{1,1} pulse with $\beta = 8/t_p$ and $\Delta f = 110$ MHz and a pulse length of (a) 100 ns, (b) 200 ns and (c) 400 ns. A 400 ns calculated pump excitation profiles next to the observer pulse excitation profiles is shown in (d).

It is expected that steeper excitation flanks lead to a smaller overlap with the observer pulses and therefore a smaller effect on the echo intensity. Despite this is the case here as well (Fig. 14d), the overlap is not reduced completely and 400 ns pulse still has some remaining spectral overlap with the observer pulses. We assume that the contradictive findings concerning the echo intensity here are caused by this remaining small overlap. It could become more perturbing for longer pulses as the overall energy of the pulses increases with the pulse length and therefore potential disturbances might be enhanced. A measurement with an larger offset between the pulses at 130 MHz shows that the echo decrease is indeed reduced (Fig. S13b) when the overlap gets smaller. Despite leading to a higher echo intensity, such a high offset is not favourable for nitroxode-nitroxide DEER, because of the limited width of the nitroxide spectrum.

S14 The influence of the B_1 field strength on chirp pulses

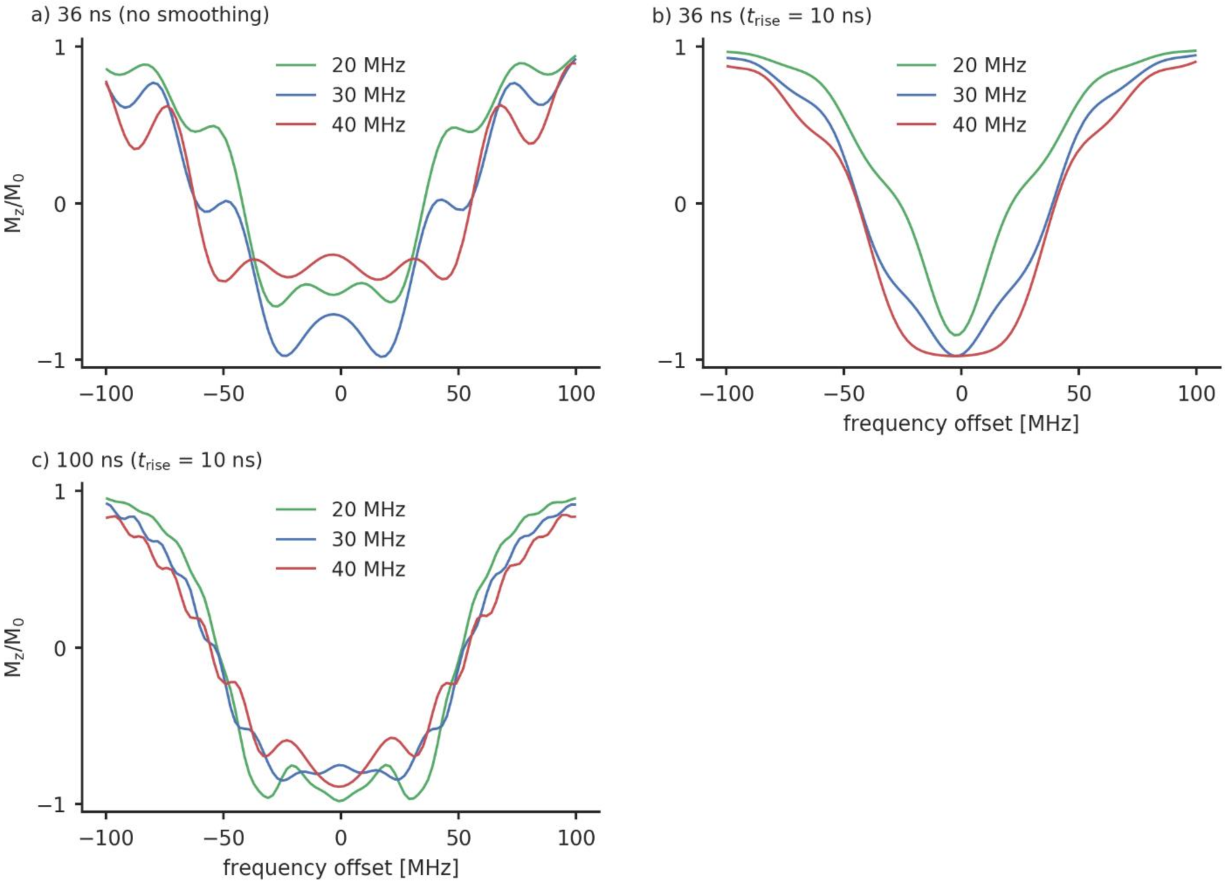

Figure S15: The inversion profiles of different chirp pulses with (a) a pulse length of 36 ns and no quarter sine smoothing, (b) a pulse length of 36 ns and a quarter sine smoothing with t_r = 10 ns and (c) a pulse length of 100 ns and a quarter sine smoothing with t_r = 10 ns. The frequency width of all pulses is Δf = 120 MHz.

S15 Comparison of bandwidth compensated and non-bandwidth compensated pulses

We tested the performance of a bandwidth compensation for HS{1,6}, WURST, chirp and HS{1,1} pump pulses. The observer pulses were rectangular with an offset of 90 MHz and an observer π pulse length of 28 ns. We estimated the effect of bandwidth compensation with the help of the η_{2p} parameter. For WURST and chirp pulses, a bandwidth compensation lead to an improvement of 3.0 % and 3.2 %. However, for HS{1,6} and HS{1,1} pulses, we observed a decrease of 10.5% and 2.6% (data not shown). As a bandwidth compensation requires a measurement of the resonator profile before each DEER measurement and did not always result in an increase in performance, we decided to stick to pulses without bandwidth compensation.

S16 The MNR for rectangular and Gaussian pump pulses evaluated up to 2 µs

Table S7: MNR for a rectangular pump pulse and different rectangular observer pulses. The pump pulses had a length of 16 ns. The MNR has been evaluated up to 2 µs.

f_{obs} [GHz]	Obs. Amp. [%]	t_{π} [ns]	MNR	Mod. depth λ
**33.91**	100	28	41	0.32
	60	32	41	0.32
**33.93**	100	28	40	0.31
	60	32	44	0.31

Table S8: MNR for a Gaussian pump pulse and different Gaussian observer pulses. The pump pulses had a length of 34 ns. The MNR has been evaluated up to 2 µs.

f_{obs} [GHz]	Obs. Amp. [%]	t_{π} [ns]	MNR	Mod. depth λ
**33.91**	100	56	50	0.31
	60	74	42	0.29
**33.93**	100	56	53	0.31
	60	74	48	0.31

S17 The MNR for broadband pump pulses evaluated up to 2 μs

Table S9: MNR for the different broadband shaped pulses with a rectangular observer pulse. The MNR has been evaluated up to 2 μs.

f_{obs} [GHz]	Obs. Amp. [%]	Pump pulse	t_π [ns]	Δf [MHz]	Offset [MHz]	MNR	Mod. depth λ
**33.91**	100	HS{1,6} ($\beta = 8/t_p$)	100	110	90	58	0.63
		WURST (n=6)	100	160	90	60	0.64
		Chirp ($t_r = 30$ ns)	36	120	90	52	0.50
		HS{1,1} ($\beta = 6/t_p$)	100	110	90	64	0.57
	60	HS{1,6} ($\beta = 10/t_p$)	100	110	90	59	0.62
		WURST (n=6)	100	160	90	63	0.63
		Chirp ($t_r = 30$ ns)	100	160	90	57	0.66
		HS{1,1} ($\beta = 6/t_p$)	100	110	90	53	0.57
**33.93**	100	HS{1,6} ($\beta = 10/t_p$)	100	110	100	60	0.57
		WURST (n=6)	100	160	100	63	0.62
		Chirp ($t_r = 10$ ns)	36	120	90	57	0.47
		HS{1,1} ($\beta = 6/t_p$)	100	110	90	65	0.53
	60	HS{1,6} ($\beta = 10/t_p$)	100	110	90	64	0.60
		WURST (n=6)	100	120	100	59	0.57
		Chirp ($t_r = 10$ ns)	36	120	90	58	0.47
		HS{1,1} ($\beta = 8/t_p$)	100	90	80	58	0.48

Table S10: MNR for the different broadband shaped pulses with a Gaussian observer pulse. The MNR has been evaluated up to 2 μs. The chirp pulse where no t_r time is specified is a pulse without the quartersine smoothing.

f_obs [GHz]	Obs. Amp. [%]	Pump pulse	t_π [ns]	Δf [MHz]	Offset [MHz]	MNR	Mod. depth λ
**33.91**	100	HS{1,6} ($\beta = 10/t_p$)	100	90	90	65	0.60
		WURST (n=6)	100	160	90	64	0.64
		Chirp ($t_\mathrm{r} = 10$ ns)	36	120	80	59	0.50
		HS{1,1} ($\beta = 6/t_p$)	100	110	90	68	0.58
	60	HS{1,6} ($\beta = 8/t_p$)	100	110	90	71	0.63
		WURST (n=6)	100	160	100	67	0.63
		Chirp ($t_r = 10$ ns)	100	120	90	59	0.48
		HS{1,1} ($\beta = 6/t_p$)	100	110	90	63	0.58
**33.93**	100	HS{1,6} ($\beta = 10/t_p$)	100	90	90	73	0.57
		WURST (n=6)	100	160	100	72	0.62
		Chirp (no smoothing)	36	120	80	65	0.49
		HS{1,1} ($\beta = 8/t_p$)	100	110	90	71	0.52
	60	HS{1,6} ($\beta = 10/t_p$)	100	110	90	82	0.61
		WURST (n=6)	100	160	90	73	0.63
		Chirp ($t_r = 9$ ns)	36	120	80	63	0.47
		HS{1,1} ($\beta = 8/t_p$)	100	110	80	74	0.52

S18 The MNR of the diluted sample evaluated up to 7 µs

Table S11: MNR for the different broadband shaped pulses with a Gaussian observer pulse. The MNR has been evaluated up to 2 µs. The chirp pulse where no t_r time is specified is a pulse without the quartersine smoothing.

f_{obs} [GHz]	Obs. Amp. [%]	Pump pulse	t_π [ns]	Δf [MHz]	Offset [MHz]	MNR	Mod. depth λ
**33.93**	100	HS{1,6} ($\beta = 10/t_p$)	100	90	90	61	0.55
		WURST (n=6)	100	160	100	54	0.59
		Chirp (no smoothing)	36	120	80	58	0.46
		HS{1,1} ($\beta = 8/t_p$)	100	110	90	65	0.47
	60	HS{1,6} ($\beta = 10/t_p$)	100	110	90	59	0.56
		WURST (n=6)	100	160	90	53	0.58
		Chirp (t_r = 9 ns)	36	120	80	54	0.43
		HS{1,1} ($\beta = 8/t_p$)	100	110	80	55	0.46

S19 Correlation between the background density and the modulation depth

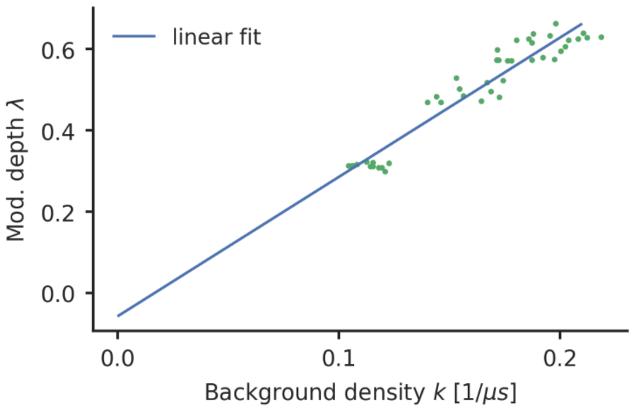

Figure S16: The correlation between the modulation depth and the background. Each dot represents a DEER trace that has been measured in the course of this study. Theoretically, the modulation depth and the background density should lie on a line through the origin. This is in fact roughly the case. The determination of the background density k seems to give a rather large error, which causes the deviations from the expected result. The fitted line has a slope of 3.43 µs and an x-axis distance of -0.06.

S20 Background decay of the DEER traces

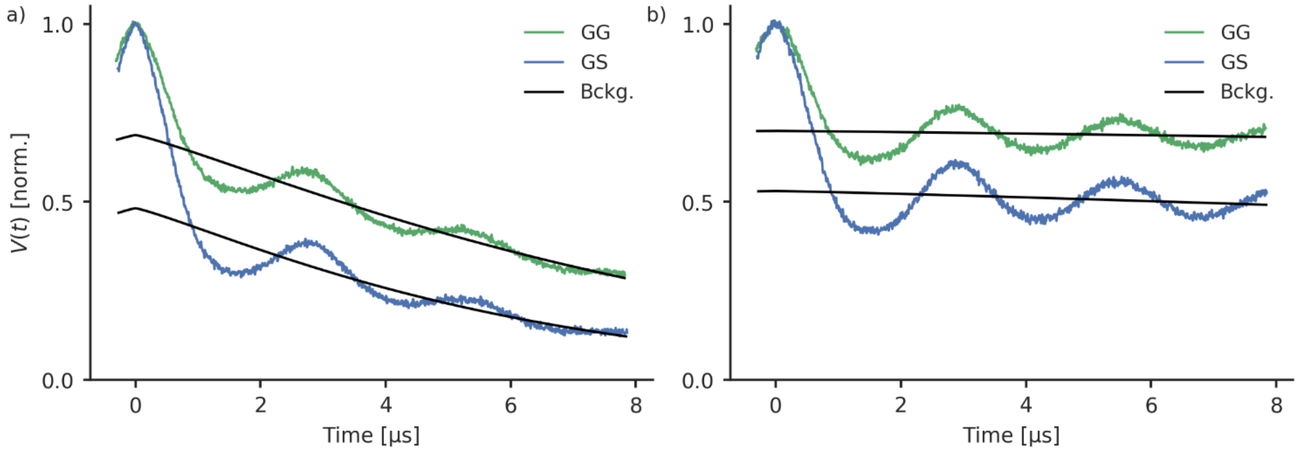

Figure S17: The (normalised) experimental raw data of the sample with a 80 µM (a) and 30 µM (b) ligand concentration. The settings for GG (green) were performed with a 100 %pulse amplitude and a 70 MHz offset. For GS (blue), the observer pulses were at a frequency of 70 MHz offset from the centre of the resonator. The pump pulses were HS{1,1} pulses, with the parameters $\beta = 8/t_p$, $t_p = 100$ ns, $\Delta f = 110$ MHz and an offset from the observer pulse of 90 MHz. Note that the acquisition time for the sample with lower concentration was longer in order to reach a similar noise level for both cases. The corresponding form factors are depicted in Fig. S11.

S21 Calculation of the background-dependent performance of broadband shaped pulses

To estimate the influence of broadband shaped pulses for different maximum distances and concentrations, we performed some analytical calculations. The background decay reduces the echo-intensity and therefore decreases the signal-to-noise ratio towards the end of the DEER trace. Whereas the measured trace $V(t)$ has a constant noise level σ_0, the background corrected form factor has an increasing noise level towards the end:

$$\sigma(t) = \sigma_0 \exp(kt), \tag{2}$$

where $\sigma(t)$ is the noise of the form factor and k is the background density. Here, we assumed a 3D background. As discussed in the main text, the form factor is truncated at a time $t_{\text{truncation}}$ to exclude the later part. An integration from $t = 0$ to $t = \tau_{\text{truncation}}$, with $\tau_{\text{truncation}}$ as the dipolar evolution time, yields the average noise in the form factor

$$\sqrt{< \sigma^2 >} = \sigma_0 \sqrt{\frac{1}{2k\tau_{\text{truncation}}} (\exp(2k\tau_{\text{truncation}}) - 1)}. \tag{3}$$

The modulation-to-noise (MNR) as the ratio of the modulation depth λ and the average noise is then described by:

$$\text{MNR} = \frac{\lambda}{\sigma_0} \sqrt{\frac{2k\tau_{\text{truncation}}}{\exp(2k\tau_{\text{truncation}}) - 1}}. \tag{4}$$

As both the modulation depth λ and the background density k directly depend on the inversion efficiency, a linear dependence can be expected between them. Indeed, we experimentally found an approximately linear correlation between them (Fig. S16). Whereas the η_{2P} value captures a decrease in echo intensity it will miss the effect of a larger background decay. We chose exemplary parameters that resembled our experimental findings. For the modulation depth, we assumed an increase from 30 % to 50 %, which corresponds to the modulation depths that we found for rectangular and the best HS{1,1} pump pulse. For the density of the background we assumed an increase about the same factor: $k_{\text{S}} = \frac{5}{3} k_{\text{R}}$ with k_{S} as the background density for the broadband shaped pulse and k_{R} as the background density for the rectangular pulses. As the sample with a concentration of 80 µM of doubly-labelled ligand had a background density k_{R} of approximately 0.1 with rectangular pulses, we tested k_{R} –values from 0 to 0.15 1/µs to keep it in a realistic range. According to equation (5) this will give an MNR increase of

$$\text{MNR}_{\text{increase}} = \frac{5}{3} \sqrt{\frac{5\,(\exp(2k_{\text{R}}\tau_{\text{truncation}}) - 1)}{3\,(\exp(\frac{10}{3}k_{\text{R}}\tau_{\text{truncation}}) - 1)}}. \tag{5}$$

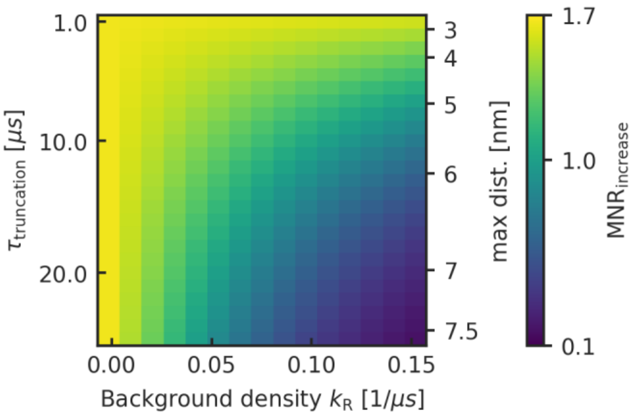

Figure S18: The MNR-ratio of adiabatic and rectangular pulses as a function of the background density (with rectangular pulses) and the $\tau_{\mathrm{truncation}}$-time. The corresponding maximum distance according to equation (13) of the main text is also depicted. As the background density reflects the concentration the x-axis is a measure for the concentration of the spin centres. In our sample with 80 µM, we had a background density k_R of 0.1 with rectangular pulses.

Figure S18 shows that the performance of shaped pulses can heavily depend on the circumstances of the measurement. For a maximum distance below 4 nm ($\tau_{\mathrm{truncation}} \approx 5$ µs), a MNR increase can be expected for all realistic concentration ranges. This is not the case if a longer distance shall be detected. For maximum distances around 5 nm, the MNR increase goes to 1 for high background densities of k_R = 0.15 1/µs, which corresponds to very high concentrations > 100 µM. Typical concentrations for DEER measurements are around 50 µM, which here corresponds to a k_R ≈ 0.06 1/µs. For this concentration, a significant increase in the MNR can only be expected up to a truncation time of $\tau_{\mathrm{truncation}}$ = 10 µs, which is equal to a maximum distance of approximately 6 nm.

As broadband shaped pulses are particularly interesting for long distances, the calculations were performed up to a rather long truncation time of 25 µs (maximum distance of approximately 7.5 nm). For distances in the range > 6 nm, only with concentrations in the range of 10-30 µM (k_R ≈ 0.01-0.04 1/µs) a significant increase in the MNR due to broadband shaped pump pulses can be expected. The MNR increase drops quickly when higher concentrations are used. For a maximum distance of 7.5 nm and for concentrations over approximately 40 µM no increase can be expected any more due to broadband shaped pulses. If a concentration of 80 µM is used, the MNR is about to decrease to roughly 40 % when switching to broadband shaped pulses. It is known that diluting the sample is favourable if long distances shall be detected because it increases the phase memory time of the echo (Schmidt et al., 2016). When broadband shaped pump pulses, the higher background decay adds an additional point for carefully choosing the concentration of the sample and it seems to be advisable to avoid high concentrations.

S22 Comparison of the resonator profiles

Figure S19 shows the resonator profiles of the measurement of the sample with the high and the low concentration. The B_1 strengths that have been achieved for the sample with the low concentration were a bit lower.

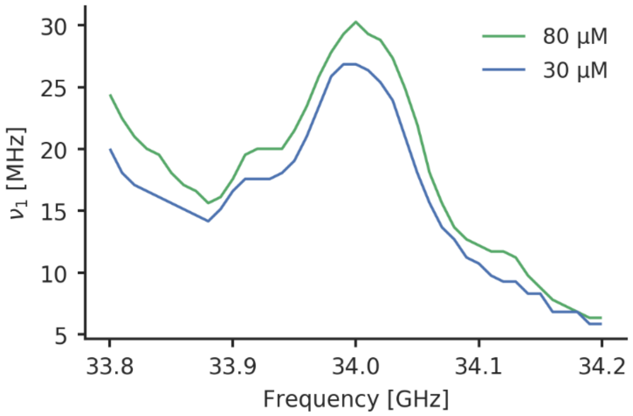

Figure S19: Resonator profiles for samples with an 80 µM concentration (green) where all optimisation measurements have been performed and for the sample with a 30 µM concentration (blue).

S23 Supporting Information References

Doll, A. and Jeschke, G.: Fourier-transform electron spin resonance with bandwidth-compensated chirp pulses, J. Magn. Reson., 246, 18–26, doi:10.1016/j.jmr.2014.06.016, 2014.

Fábregas Ibáñez, L. and Jeschke, G.: Optimal background treatment in dipolar spectroscopy, Phys. Chem. Chem. Phys., 22(4), 1855–1868, doi:10.1039/C9CP06111H, 2020.

Jeschke, G.: DEER Distance Measurements on Proteins, Annu. Rev. Phys. Chem., 63(1), 419–446, doi:10.1146/annurev-physchem-032511-143716, 2012.

Jeschke, G., Chechik, V., Ionita, P., Godt, A., Zimmermann, H., Banham, J., Timmel, C. R., Hilger, D. and Jung, H.: DeerAnalysis2006—a comprehensive software package for analyzing pulsed ELDOR data, Appl. Magn. Reson., 30(3), 473–498, doi:10.1007/BF03166213, 2006.

Schmidt, T., Wälti, M. A., Baber, J. L., Hustedt, E. J. and Clore, G. M.: Long Distance Measurements up to 160 Å in the GroEL Tetradecamer Using Q-Band DEER EPR Spectroscopy, Angew. Chem. Int. Ed., 55(51), 15905–15909, doi:10.1002/anie.201609617, 2016.

Stoll, S. and Schweiger, A.: EasySpin, a comprehensive software package for spectral simulation and analysis in EPR, J. Magn. Reson., 178(1), 42–55, doi:10.1016/j.jmr.2005.08.013, 2006.

Tait, C. E. and Stoll, S.: Coherent pump pulses in Double Electron Electron Resonance spectroscopy, Phys. Chem. Chem. Phys., 18(27), 18470–18485, doi:10.1039/C6CP03555H, 2016.
